# Multi-omics analysis and experimental validation of the value of monocyte-associated features in prostate cancer prognosis and immunotherapy

**DOI:** 10.3389/fimmu.2024.1426474

**Published:** 2024-06-14

**Authors:** YaXuan Wang, Chao Li, JiaXing He, QingYun Zhao, Yu Zhou, HaoDong Sun, HaiXia Zhu, BeiChen Ding, MingHua Ren

**Affiliations:** ^1^ Department of Urology, The First Affiliated Hospital of Harbin Medical University, Harbin, China; ^2^ Department of General, Visceral, and Transplant Surgery, Ludwig-Maximilians-University Munich, Munich, Germany; ^3^ Clinical Laboratory, Tumor Hospital Affiliated to Nantong University, Nantong, China

**Keywords:** prognosis, monocyte, machine learning, PRAD, multi-omics analysis

## Abstract

**Background:**

Monocytes play a critical role in tumor initiation and progression, with their impact on prostate adenocarcinoma (PRAD) not yet fully understood. This study aimed to identify key monocyte-related genes and elucidate their mechanisms in PRAD.

**Method:**

Utilizing the TCGA-PRAD dataset, immune cell infiltration levels were assessed using CIBERSORT, and their correlation with patient prognosis was analyzed. The WGCNA method pinpointed 14 crucial monocyte-related genes. A diagnostic model focused on monocytes was developed using a combination of machine learning algorithms, while a prognostic model was created using the LASSO algorithm, both of which were validated. Random forest and gradient boosting machine singled out CCNA2 as the most significant gene related to prognosis in monocytes, with its function further investigated through gene enrichment analysis. Mendelian randomization analysis of the association of HLA-DR high-expressing monocytes with PRAD. Molecular docking was employed to assess the binding affinity of CCNA2 with targeted drugs for PRAD, and experimental validation confirmed the expression and prognostic value of CCNA2 in PRAD.

**Result:**

Based on the identification of 14 monocyte-related genes by WGCNA, we developed a diagnostic model for PRAD using a combination of multiple machine learning algorithms. Additionally, we constructed a prognostic model using the LASSO algorithm, both of which demonstrated excellent predictive capabilities. Analysis with random forest and gradient boosting machine algorithms further supported the potential prognostic value of CCNA2 in PRAD. Gene enrichment analysis revealed the association of CCNA2 with the regulation of cell cycle and cellular senescence in PRAD. Mendelian randomization analysis confirmed that monocytes expressing high levels of HLA-DR may promote PRAD. Molecular docking results suggested a strong affinity of CCNA2 for drugs targeting PRAD. Furthermore, immunohistochemistry experiments validated the upregulation of CCNA2 expression in PRAD and its correlation with patient prognosis.

**Conclusion:**

Our findings offer new insights into monocyte heterogeneity and its role in PRAD. Furthermore, CCNA2 holds potential as a novel targeted drug for PRAD.

## 1 Introduction

Based on 2024 U.S. cancer statistics, prostate adenocarcinoma (PRAD) now surpasses lung cancer as the most common cancer among men (1). In China, recent data from the China National Cancer Center in 2022 revealed that PRAD incidence rates have exceeded those of kidney and bladder tumors based on 2016 data from 487 tumor registries nationwide (2). The incidence of PRAD has been on the rise in recent years due to economic and social development and increased life expectancy. Options for treating PRAD currently consist of radical radiotherapy, radical prostatectomy, chemotherapy, and androgen deprivation therapy, customized based on the progression of the individual patient’s illness (3). Despite advancements in PRAD treatment, the 5-year survival rate for patients remains relatively low (4). Therefore, it is crucial to identify potential prognostic markers and assess therapeutic targets to improve the prognosis of PRAD patients.

Monocytes are vital components of the innate immune system and are indispensable for defending against foreign invaders (5). There are three primary subpopulations of monocytes: classical, nonclassical, and intermediate monocytes (6). Monocytes first mature into classical monocytes in the bone marrow, followed by differentiation into nonclassical monocytes in the bloodstream, with an intermediate monocyte phase in between. Numerous studies have shown that monocytes play a direct role in immune responses by initiating cell death and phagocytosis (7). Additionally, monocytes can engage with T cells and natural killer cells, impacting tumor progression by producing chemokines (8). Moreover, monocytes have the capability to transform into various immune cells such as tumor-associated macrophages and dendritic cells, critical components of the immune system that actively promote tumor growth and spread (9). Tumor-infiltrating immune cells play a crucial role in the pathogenesis of PRAD. Recent research indicates that prognostic markers linked to M2 macrophages can forecast biochemical recurrence in patients with PRAD (10). Furthermore, elevated levels of macrophages in prostate biopsies have been correlated with disease progression following hormone therapy (11). Moreover, there is evidence to suggest that circulating monocyte levels could serve as a biomarker for metastatic PRAD, indicating a notably unfavorable prognosis (12). Integrated multi-omics, machine learning, and artificial intelligence are being more frequently utilized in the field of medicine (13–17). It is essential to conduct further analysis on levels of tumor-infiltrating immune cells and identify genes related to immune cell infiltration using multi-omics and machine learning techniques to enhance the accuracy of diagnosis and treatment for PRAD. The objective of our study is to enhance researchers’ comprehension of the mechanisms underlying tumor immune infiltration, progress in immunotherapy for PRAD patients, and offer novel insights for clinical immunotherapy.

The significance of immune cell infiltration in tumors and the exploration of its potential regulatory genes have been acknowledged based on existing research. The CIBERSORT algorithm provides a convenient method for evaluating immune cell infiltration levels in PRAD. By utilizing this algorithm, we calculated the infiltration levels of immune cells in TCGA-PRAD samples and grouped the samples accordingly. Our analysis revealed that only the infiltration level of monocytes significantly correlated with the prognosis of PRAD patients. Using the weighted correlation network analysis (WGCNA) method, we have discovered prognostic differential genes associated with monocytes in the PRAD dataset samples from the cancer genome atlas (TCGA) database. These genes exhibit correlations with patient stage, Gleason score, and PSA score. Subsequently, we developed diagnostic and prognostic models using various machine learning techniques, yielding positive results. By analyzing the TCGA-PRAD and GSE16560 datasets, CCNA2 emerged as the most promising prognostic gene related to monocytes in PRAD. Furthermore, we delved into the function of CCNA2 and its potential interactions with therapeutic drugs for PRAD. In conclusion, our study lays the groundwork for understanding the impact of monocytes on the prognosis of PRAD patients and identifies a novel drug target for PRAD treatment.

## 2 Materials and methods

### 2.1 Data acquisition

The TCGA database provided data on 52 normal prostate samples and 498 PRAD samples. The monocyte-related gene diagnostic model was validated using the GSE62872 and GSE32571 datasets, while the GSE16560 dataset was used for the prognostic model of monocyte-related genes. In addition, 60 cases of prostate cancer tissue and paired para-cancerous tissue were obtained from Shanghai Outdo Biotech Company. The patients included in the tissue chip study underwent surgery between January 2011 and December 2014, with a follow-up period extending from November 2021, covering a span of 6 to 10 years.

### 2.2 Constructing diagnostic and prognostic models

Use multiple machine learning algorithms to combine into more than one hundred algorithm combinations to develop the best PRAD diagnostic model (18). The training set comprised the TCGA-PRAD data set, with validation sets GSE62872 and GSE32571. Area under curve (AUC) values were calculated for each algorithm combination, and the combination with the highest average AUC was selected as the best. The prognostic model was based on the least absolute shrinkage and selection operator (LASSO) regression algorithm and evaluated using 10-fold cross-validation in R software with the glmnet package (19, 20).

### 2.3 Functional analysis of candidate genes

The gene set cancer analysis (GSCA) and CancerSEA databases were used to analyze the functions of monocyte-related genes (21, 22). To better understand the oncogenic role of target genes, the ClusterProfiler package in R was used to analyze the potential functions of CCNA2 and enrich the Kyoto encyclopedia of genes and genomes (KEGG) pathway. The R packages “clusterProfiler” was utilized for the GSEA enrichment analysis of genes (23).

### 2.4 Analysis of the correlation between CCNA2 and immune cell infiltration

The GSCA database was utilized to examine the relationship between CCNA2 and monocytes. Furthermore, we investigated the correlation between CCNA2 and markers of monocytes using the TCGA-PRAD dataset. Additionally, the TISCH2 database was employed to analyze the association between CCNA2 and immune cell infiltration (24).

### 2.5 Immunohistochemical staining analysis of CCNA2 expression in PRAD tissues

The prostate cancer tissue chip was initially placed in an 85°C oven for 20 minutes, followed by soaking in xylene solution for 20 minutes for dewaxing. Subsequently, the tissue chips underwent a series of hydration steps involving immersion in 100%, 95%, 80%, and 70% ethanol for 2 minutes each. The tissue chip was then treated with citric acid solution and subjected to boiling in a pressure cooker for antigen retrieval, followed by cooling in ice water to reach room temperature. The chip was then rinsed with PBS, circled with a histochemistry pen, sealed with hydrogen peroxide solution, and cleaned with PBS. Next, CCNA2 antibody (BOSTER, PB9424) was applied dropwise to cover the tissue chip, which was left at room temperature for 2 hours. Post-reaction, the chip was rinsed with PBS and the immunohistochemistry secondary antibody was added dropwise, left for 20 minutes, and then cleaned with PBS. Finally, the tissue chip underwent DAB color development, dehydration in a series of ethanol solutions, sealing, and microscopic examination to conclude the experiment. The immunostaining intensity score ranges from 0 to 3, where 0, 1, 2, and 3 represent no reaction, weak reaction, moderate reaction, and strong reaction, respectively. Following this, a scale based on the proportion of positive staining is applied, with scores of 1, 2, 3, and 4 corresponding to 0%-25%, 26%-50%, 51%-75%, and 76%-100%, respectively. The final expression score is determined by multiplying the staining intensity score and the staining proportion score. This calculation results in a score ranging from 0 to 5, indicating low expression, and a score from 6 to 12, indicating high expression.

### 2.6 Mendelian randomization analysis

The Mendelian randomization analysis in this study investigated the impact of monocytes on prostate cancer patients using the MRBASE website (25). The exposure factor selected was HLA DR++ monocyte %monocyte (ebi-a-GCST90001475) from the MR Base GWAS catalog, with prostate cancer (EBI-A-GCST006085) as the outcome. The analysis criteria included a minimum LD Rsq value of 0.8, a MAF threshold of 0.01, and the exclusion of palindromic SNPs. Various methods such as MR Egger, Weighted median, Weighted mode, Simple mode, and Inverse variance weighted were employed for the analysis.

### 2.7 Statistical analyses

The level of immune cell infiltration and prognosis of TCGA-PRAD patients, along with the prognostic analysis of CCNA2 in prostate cancer tissue chips used in our experiments, were statistically analyzed using the Log-rank test. The prognostic analysis of ACSM3 and CCNA2 in TCGA-PRAD and GSE16560 datasets was conducted through COX regression. All correlation analyses in this study were performed using the Spearman method. Furthermore, the expression of monocyte-related genes at different stages, Gleason scores, and PSA scores in the TCGA-PRAD dataset, as well as the expression differences of CCNA2 in prostate cancer tissue chips, were analyzed using the Wilcoxon rank sum test.

## 3 Results

### 3.1 Analysis of the correlation between the level of immune cell infiltration and the prognosis of PRAD patients

**Figure 1:**
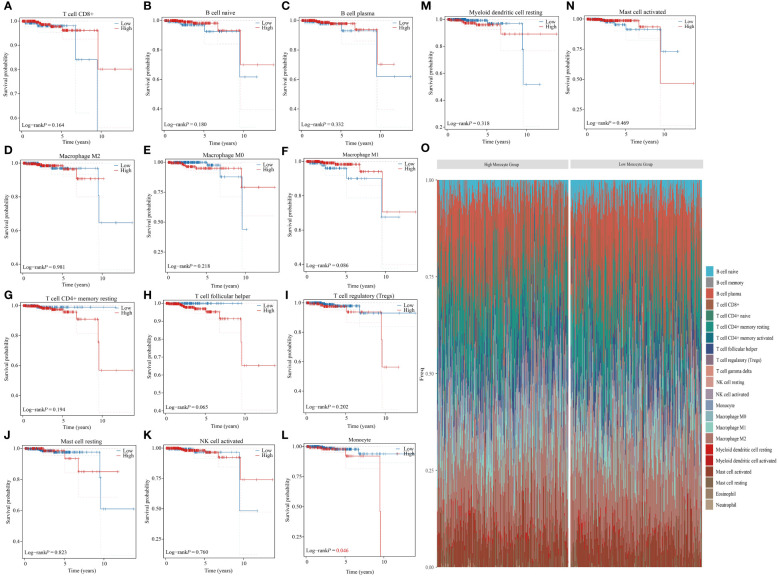
Monocyte infiltration correlates with prognosis in PRAD patients. **(A–N)** Analyzing the correlation between different immune cell infiltrations and prognosis in PRAD patients. **(O)** Percentage frequency of different tumor infiltrating immune cells in PRAD samples.

The tumor immune microenvironment consists of tumor cells, immune cells, signaling molecules, extracellular matrix, and unique physical and chemical characteristics (26). This microenvironment significantly impacts tumor diagnosis, survival rates, and treatment responses. Immune cell infiltration in tumors is crucial as it can either help eliminate tumor cells or be manipulated by tumors to promote growth and metastasis (27, 28). The role of immune cells in cancer treatment and prevention, as well as their regulatory mechanisms, has garnered significant attention. An accurate understanding of the distribution and function of immune cells in tumor tissues is essential for effective treatment and prognosis assessment (29, 30). The CIBERSORT algorithm was used to calculate the proportion of 22 immune cells for each sample in the TCGA-PRAD dataset (31, 32). While the infiltration level of 8 types of immune cells in most samples was 0, our study focused on analyzing the relationship between the infiltration levels of the remaining 14 types of immune cells and the prognosis of PRAD patients. Our findings suggest that the infiltration level of monocytes is a significant factor in determining the prognosis of patients with PRAD. Specifically, a higher infiltration level of monocytes is associated with a poorer prognosis for PRAD patients (**Figures 1A–N**). Furthermore, based on monocyte infiltration levels, PRAD patient samples were classified into high and low monocyte groups. We then examined the percentage abundance of tumor-infiltrating immune cells in each sample (**Figure 1O**).

### 3.2 Screening of monocyte-associated differential genes based on the WGCNA method

**Figure 2:**
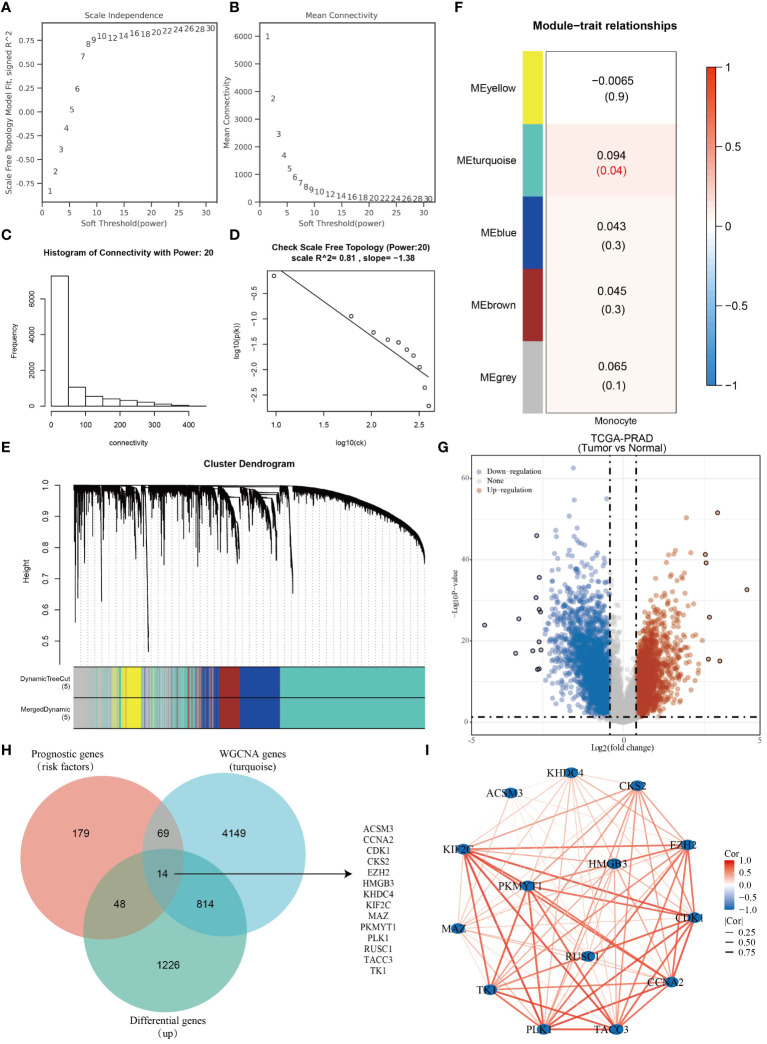
Fourteen monocyte-associated differential prognostic genes were identified. **(A–D)** WGCNA Network Construction Parameters. **(E)** Weighted co-expression network modeling based on selected power values. **(F)** Heatmap of trait module associations. **(G)** Analysis of differences in TCGA-PRAD dataset. **(H)** Venn diagram based on the intersection of TCGA-PRAD differential genes, prognostic genes and monocyte-associated genes. **(I)** Correlation network diagram of monocyte-associated prognostic differential genes.

WGCNA is an algorithm utilized for extracting module information from high-throughput expression data. Our objective was to identify genes highly correlated with monocytes in the TCGA-PRAD dataset using this algorithm. To achieve a scale-free network distribution, we carefully selected the value of the adjacency matrix weight parameter power. In our analysis, we determined the power value to be 20 (**Figures 2A–D**). Subsequently, a weighted co-expression network model was constructed based on this power value, leading to the division of the gene set into 5 modules. Notably, the gray module represents genes that do not align with any specific module and lack reference significance (**Figure 2E**). Using the Pearson correlation algorithm, we found that the turquoise module has the strongest correlation with monocytes (**Figure 2F**). We conducted differential analysis on TCGA-PRAD samples with a significance level of P < 0.05 and Log2 (Fold Change) >1.3 or Log2 (Fold Change) < -1.3 as the selection criteria. Subsequently, we generated a volcano plot to visualize the analysis outcomes (**Figure 2G**). Our findings indicated a link between high levels of mononuclear cell infiltration and poor prognosis in PRAD patients. Through the intersection of genes in the turquoise module with prognostic risk factors in the TCGA-PRAD dataset and genes highly expressed in PRAD, we identified a total of 14 monocyte-related prognostic differential genes (**Figure 2H**). Importantly, these 14 genes were found to be positively correlated with each other (**Figure 2I**).

### 3.3 Functional analysis of monocyte-related prognostic differential genes

**Figure 3:**
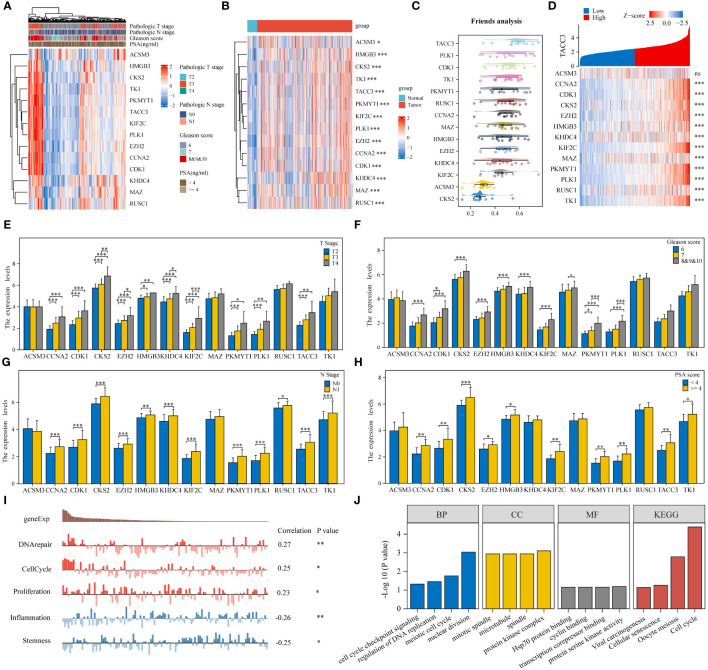
Monocyte-associated prognostic differential genes play an important role in PRAD. **(A)** Heatmap of monocyte-associated prognostic differential gene expression in different pathologic parameters. **(B)** Heatmap of monocyte-associated prognostic differential gene expression in PRAD and normal tissues. **(C)** Friends analysis explores key genes in monocyte-associated genes. **(D)** Heatmap of co-expression in monocyte-associated genes. **(E–H)** Histogram of monocyte-related gene expression in different clinicopathologic parameters. **(I, J)** Functional analysis of monocyte-related genes. *p< 0.05, **p< 0.01, ***p< 0.001.

The study initially examined the relationship between 14 genes and clinicopathological characteristics of PRAD patients, illustrating this correlation through a heatmap (**Figure 3A**). Additionally, expression heatmaps were generated for the 14 genes in TCGA-PRAD samples and normal prostate tissue (**Figure 3B**). Friends analysis aimed to develop a gene interaction network, leveraging network topology to assess gene importance and identify key genes. Notably, TACC3 emerged as the central gene within this network (**Figure 3C**). A co-expression network diagram was constructed with TACC3 at its core, revealing that ACSM3 exhibited no correlation with TACC3, while the remaining 13 genes showed significant correlations with TACC3 (**Figure 3D**). The expression levels of CCNA2, CDK1, CKS2, EZH2, HMGB3, KHDC4, KIF2C, PKMYT1, and PLK1 were found to vary significantly across different T stages, N stages, Gleason scores, and PSA scores in TCGA-PRAD samples (**Figures 3E–H**). Utilizing the CancerSEA database, which is tailored to decode the diverse functional states of cancer cells at a single-cell level, we investigated the functions of these 14 genes in PRAD. Our analysis revealed that these genes play roles in DNA repair, cell cycle regulation, proliferation, inflammation, and stemness (**Figure 3I**). Furthermore, through enrichment analysis, we discovered that these genes are primarily associated with cell cycle processes (**Figure 3J**).

### 3.4 Multiple machine learning combinations to build PRAD diagnostic models

**Figure 4:**
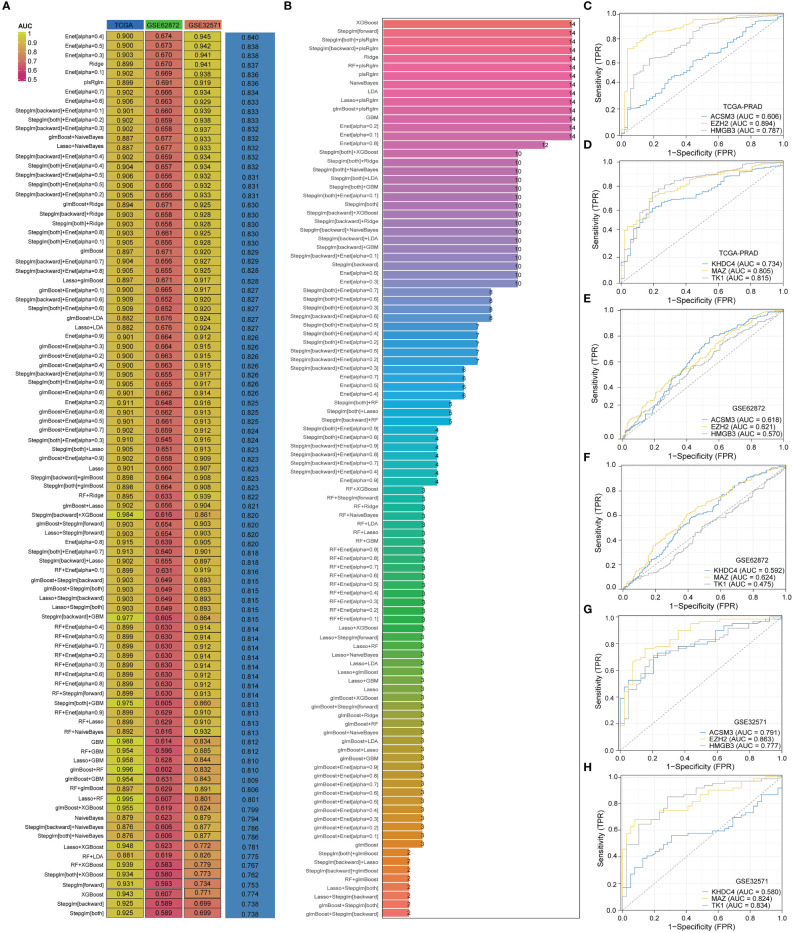
The model constructed by Enet [alpha=0.4] algorithm is the best PRAD diagnostic model. **(A)** AUC values of diagnostic models constructed with different combinations of algorithms. **(B)** Number of genes incorporated in diagnostic models constructed with different combinations of algorithms. **(C–H)** Diagnostic value of genes in diagnostic models in different datasets.

In order to develop a PRAD-related diagnosis model, we utilized three PRAD datasets: the TCGA-PRAD dataset for training, and the GSE62872 and GSE32571 datasets for validation. Out of 94 algorithm combinations, the Enet[alpha=0.4] algorithm was identified as the most effective for constructing the diagnostic model (**Figure 4A**). The AUC value for the TCGA-PRAD training set was 0.9, while the AUC values for the validation sets GSE62872 and GSE32571 were 0.674 and 0.945. The diagnostic model built by the Enet[alpha=0.4] algorithm featured six genes: ACSM3, EZH2, HMGB3, KHDC4, MAZ, and TK1 (**Figure 4B**). Additionally, ROC curves for these six genes in the TCGA-PRAD, GSE62872, and GSE32571 datasets were presented (**Figures 4C–H**). The AUC values of ACSM3, EZH2, HMGB3, KHDC4, MAZ, and TK1 in the TCGA-PRAD dataset are 0.606, 0.894, 0.787, 0.734, 0.805, and 0.815, respectively. Similarly, in the GSE62872 dataset, these values are 0.618, 0.621, 0.570, 0.624, 0.624, and 0.475. While the diagnostic potential of these genes for PRAD in the initial dataset is significant, it lacks precision. To address this, we conducted further analysis using the GSE32571 dataset as a validation set, where the AUC values for the six genes were 0.791, 0.863, 0.777, 0.580, 0.824, and 0.834.

### 3.5 Constructing prognostic model

**Figure 5:**
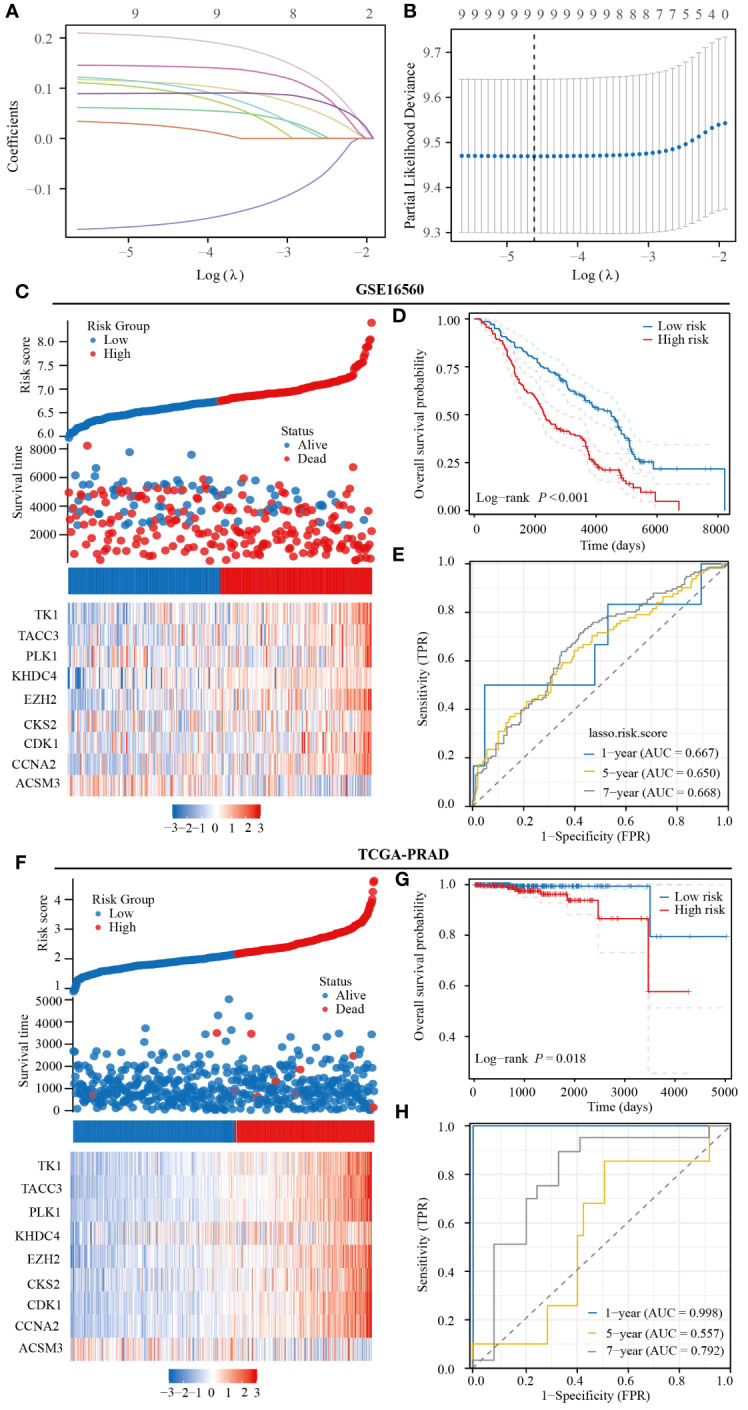
Prognostic model constructed based on monocyte-related genes has strong predictive value for the prognosis of PRAD patients. **(A, B)** Prognostic modeling based on the LASSO algorithm. **(C)** Heatmap of expression of prognostic model genes included in the GSE16560 dataset. **(D)** Prognostic differences between patients in the high- and low-risk groups in the GSE16560 dataset. **(E)** Predictive value of the GSE16560 dataset risk score for prognosis in patients with PRAD. **(F)** Heatmap of expression of prognostic model genes included in TCGA-PRAD dataset. **(G)** Prognostic differences between patients in the high- and low-risk groups in TCGA-PRAD dataset. **(H)** Predictive value of the TCGA-PRAD dataset risk score for prognosis in patients with PRAD.

To enhance the prediction accuracy of PRAD patient prognosis, we developed a prognostic model utilizing monocyte-related genes through the LASSO algorithm. This model incorporated 9 genes, with corresponding risk scores calculated as follows: ACSM3*(-0.17156) +CCNA2*(0.11148) +CDK1*(0.05883) +CKS2*(0.09723) +EZH2*(0.20259) +KHDC4*(0.10915) +PLK1*(0.02555) +TACC3*(0.14386) +TK1*(0.08982) (**Figures 5A, B**). Initial validation in the GSE16560 dataset indicated a notably poorer prognosis for patients classified in the high-risk group compared to those in the low-risk group. Additionally, our prognostic model exhibited predictive abilities for 1-year, 5-year, and 7-year prognoses of PRAD patients, with corresponding AUC values of 0.667, 0.650, and 0.668, respectively (**Figures 5C–E**). Subsequent validation in the TCGA-PRAD dataset confirmed the accuracy of our prognostic model in predicting patient outcomes, particularly for 1-year and 7-year prognoses. However, the predictive ability for the 5-year prognosis of PRAD patients was found to be moderate (**Figures 5F–H**).

### 3.6 Multiple machine learning approaches to identify monocyte-associated prognostic genes

**Figure 6:**
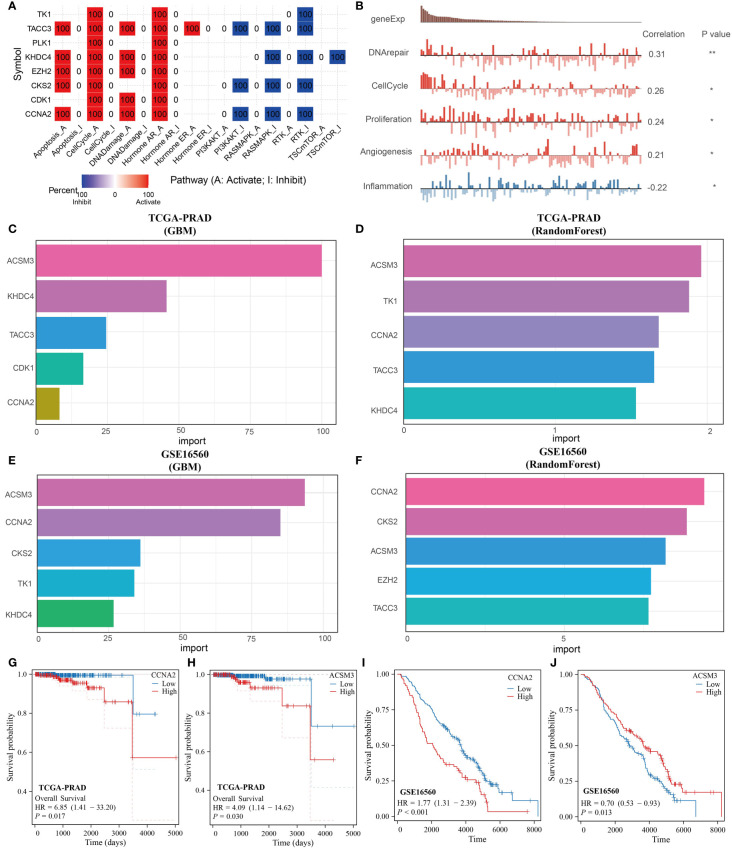
CCNA2 identified as the best prognostic gene among monocyte-associated genes. **(A, B)** Functional analysis of monocyte-related prognostic genes. **(C, D)** GBM and RandomForest algorithms to screen key prognostic genes in the TCGA-PRAD dataset. **(E, F)** GBM and RandomForest algorithms to screen key prognostic genes in the GSE16560 dataset. **(G, H)** KM curves of CCNA2 and ACSM3 in the TCGA-PRAD dataset. **(I, J)** KM curves of CCNA2 and ACSM3 in the GSE16560 dataset.

The key genes incorporated into the prognostic model were further analyzed. These genes were primarily associated with the cell cycle and activation of the hormone AR (**Figure 6A**). Validation from the CancerSEA database confirmed that these prognostic genes were linked to DNA repair, cell cycle, proliferation, angiogenesis, and inflammation (**Figure 6B**). Utilizing the GBM and Random Forest algorithms, we identified the top 5 genes most relevant to the prognosis of PRAD for display. CCNA2 and ACSM3 were found to have significant prognostic value in both the TCGA-PRAD and GSE16560 datasets (**Figures 6C–F**). Subsequently, prognostic KM curves for CCNA2 and ACSM3 were presented, revealing an opposite prognostic difference for ACSM3 in the two datasets, possibly due to insufficient sample size. However, the prognostic difference for CCNA2 in the two datasets remained consistent (**Figures 6G–J**). Thus, among monocyte-related genes, CCNA2 was identified as the gene with the highest prognostic correlation with PRAD.

### 3.7 CCNA2 is associated with monocyte infiltration in PRAD

**Figure 7:**
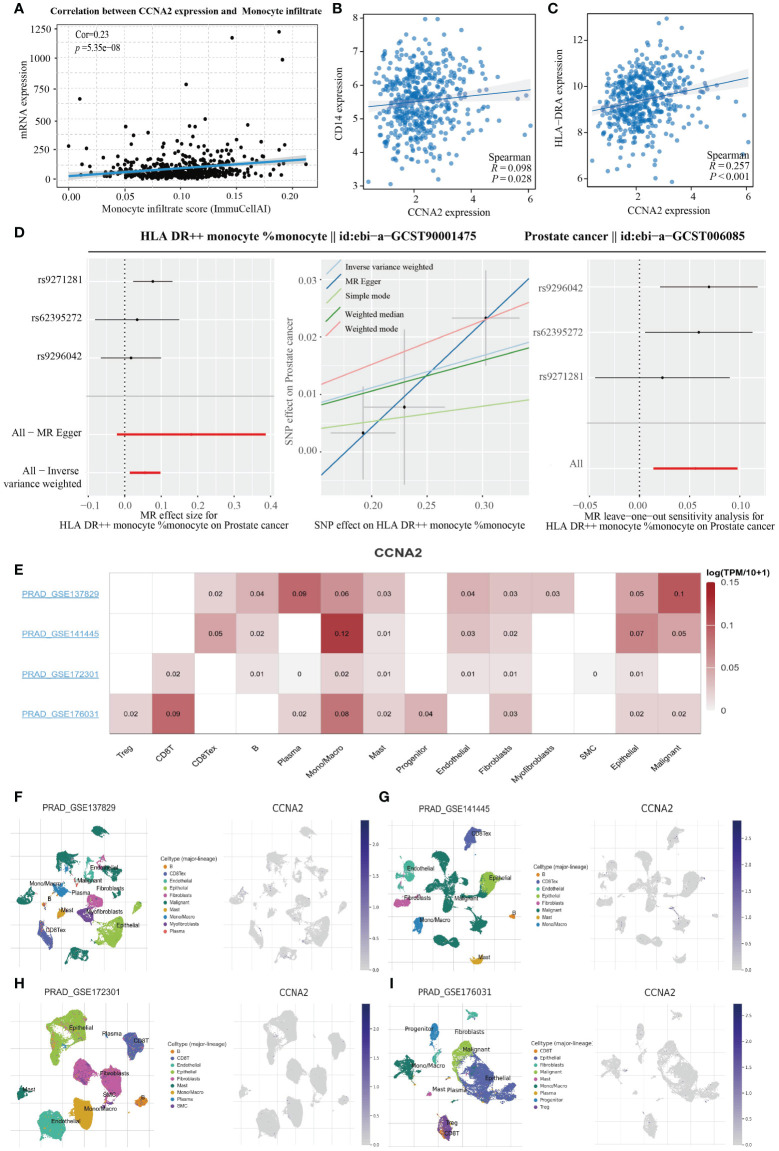
CCNA2 positively correlates with monocyte infiltration levels. **(A)** Correlation analysis of CCNA2 and monocyte infiltration levels. **(B, C)** Analysis of CCNA2 correlation with monocyte markers. **(D)** Mendelian randomization analysis of high HLA-DR expressing monocytes in relation to prostate cancer. **(E–I)** Single-cell analysis of the correlation between CCNA2 and immune cell infiltration.

In order to further investigate the relationship between the genes screened in PRAD and immune cell infiltration, we conducted an analysis on the correlation between CCNA2 expression in PRAD and monocytes using the GSCA database. Our results revealed a positive correlation between CCNA2 expression and the level of monocyte infiltration, with a correlation coefficient of 0.23 (**Figure 7A**). Furthermore, we conducted a correlation analysis on the TCGA-PRAD dataset to explore the relationship between CCNA2 expression and monocyte markers. Our findings indicated a significant association between CCNA2 and the monocyte markers CD14 and HLA-DRA (**Figures 7B, C**). In light of our research, we observed a strong correlation between CCNA2 and the monocyte marker HLA-DRA. Subsequently, we conducted further analysis to investigate the connection between monocytes expressing high levels of HLA-DR and prostate cancer using Mendelian randomization. Our results indicate that monocytes with elevated HLA-DR expression contribute to the progression of prostate cancer (**Figure 7D**). The correlation between CCNA2 and immune cell infiltration in PRAD was investigated using single cell analysis from the TISCH2 database. Our findings revealed that CCNA2 was linked to the levels of monocytes and macrophages infiltration in the GSE137829, GSE141445, GSE172301, and GSE176031 datasets (**Figures 7E–I**).

### 3.8 Gene enrichment analysis of CCNA2

**Figure 8:**
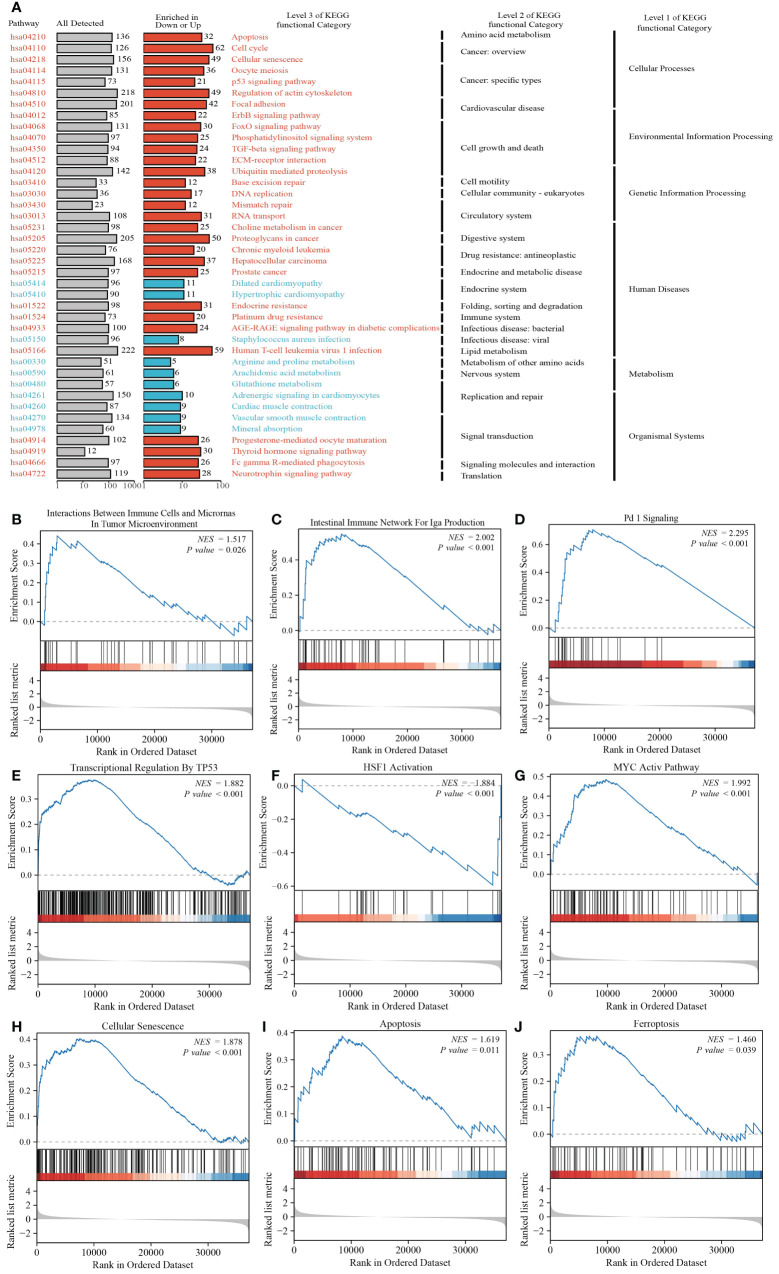
Functional analysis of CCNA2 in PRAD. **(A)** KEGG analysis of CCNA2 in PRAD. **(B–J)** GSEA analysis of CCNA2 in PRAD.

KEGG analysis revealed that CCNA2 is associated with various pathways in PRAD, including the cell cycle, Human T-cell leukemia virus 1 infection, Proteoglycans in cancer, and Regulation of actin cytoskeleton. Additionally, it is linked to pathways like p53 signaling, TGF-beta signaling, and AGE-RAGE signaling in diabetic complications (**Figure 8A**). GSEA analysis further highlighted the role of CCNA2 in the immune microenvironment of PRAD, potentially influencing immunotherapy through the PD1 signaling pathway. The association of CCNA2 with transcription factors such as P53, HSF1, and MYC was noted, although experimental validation is needed. Furthermore, CCNA2 was found to regulate PRAD cell senescence, apoptosis, and ferroptosis (**Figures 8B–J**).

### 3.9 Analysis of CCNA2 and drug affinity in metastatic PRAD

**Figure 9:**
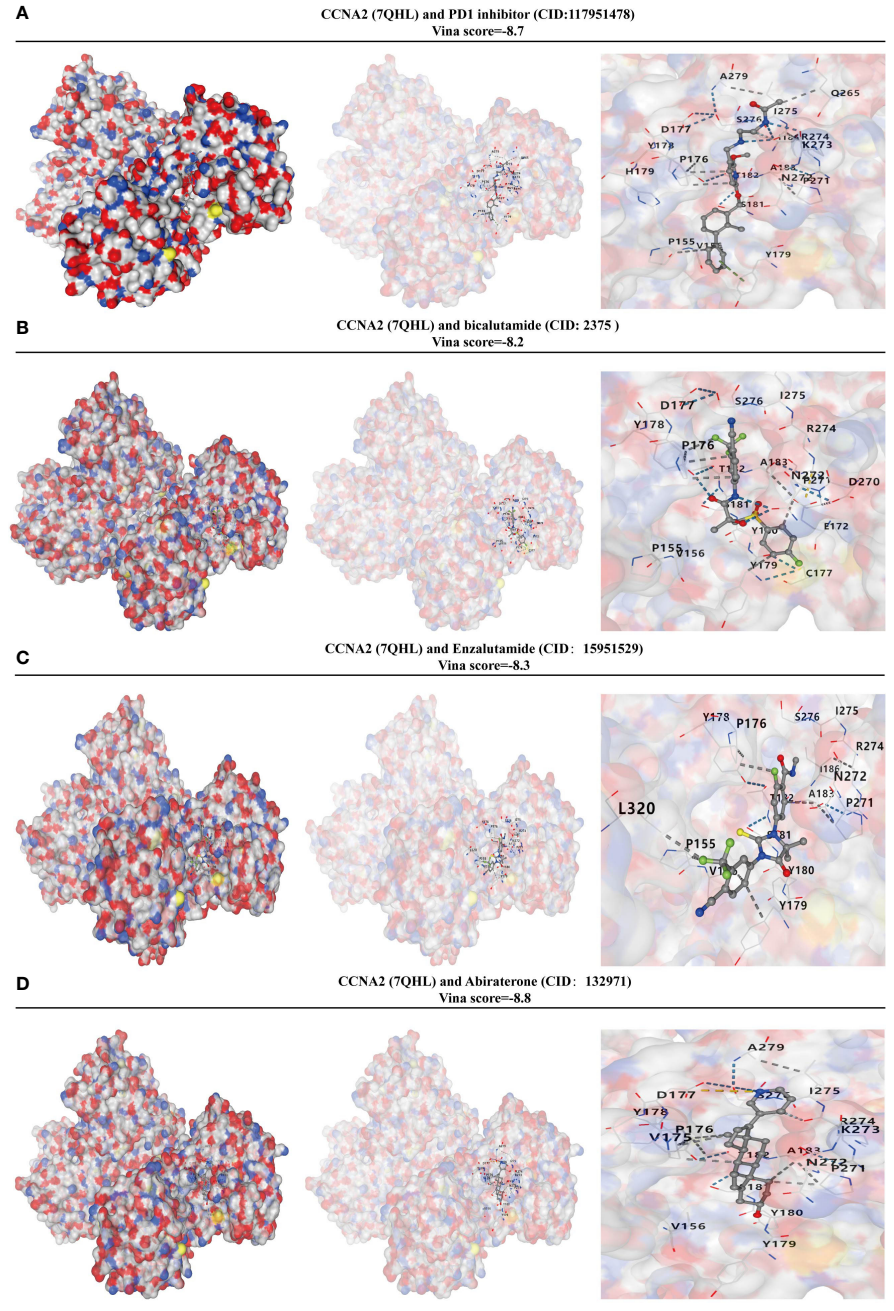
CCNA2 has a high binding capacity to PRAD-targeted drugs. **(A)** Analysis of the binding capacity of CCNA2 to PD1 inhibitors. **(B)** Analysis of the binding capacity of CCNA2 to bicalutamide. **(C)** Analysis of the binding capacity of CCNA2 to enzalutamide. **(D)** Analysis of the binding capacity of CCNA2 to abiraterone.

In order to assess the binding affinity of the key gene CCNA2 with PRAD-targeted drugs, we utilized molecular docking methods for analysis. The CB-Dock2 website, known for its molecular docking analysis capabilities, facilitated our research. The Vina score was employed to measure the binding affinity between genes and drugs. A Vina score below -5 indicates strong binding activity, with lower scores indicating higher binding activity. The results of our GSEA analysis revealed a close relationship between CCNA2 and the PD1 signaling pathway. We further investigated the molecular binding affinity of CCNA2 with PD1 inhibitors and found a strong affinity in their molecular structures. With a vina score of -8.7, indicating high binding ability (**Figure 9A**). Additionally, we examined the binding ability of targeted drugs for metastatic PRAD - Bicalutamide, enzalutamide, and abiraterone - to CCNA2. Our findings demonstrated a strong binding ability of CCNA2 to these drugs at a molecular level (**Figures 9B–D**). These results not only suggest that CCNA2 may enhance the anti-cancer effects of these drugs but also support the potential of CCNA2 as a drug target.

### 3.10 Expression and prognostic value of CCNA2 in PRAD

**Figure 10:**
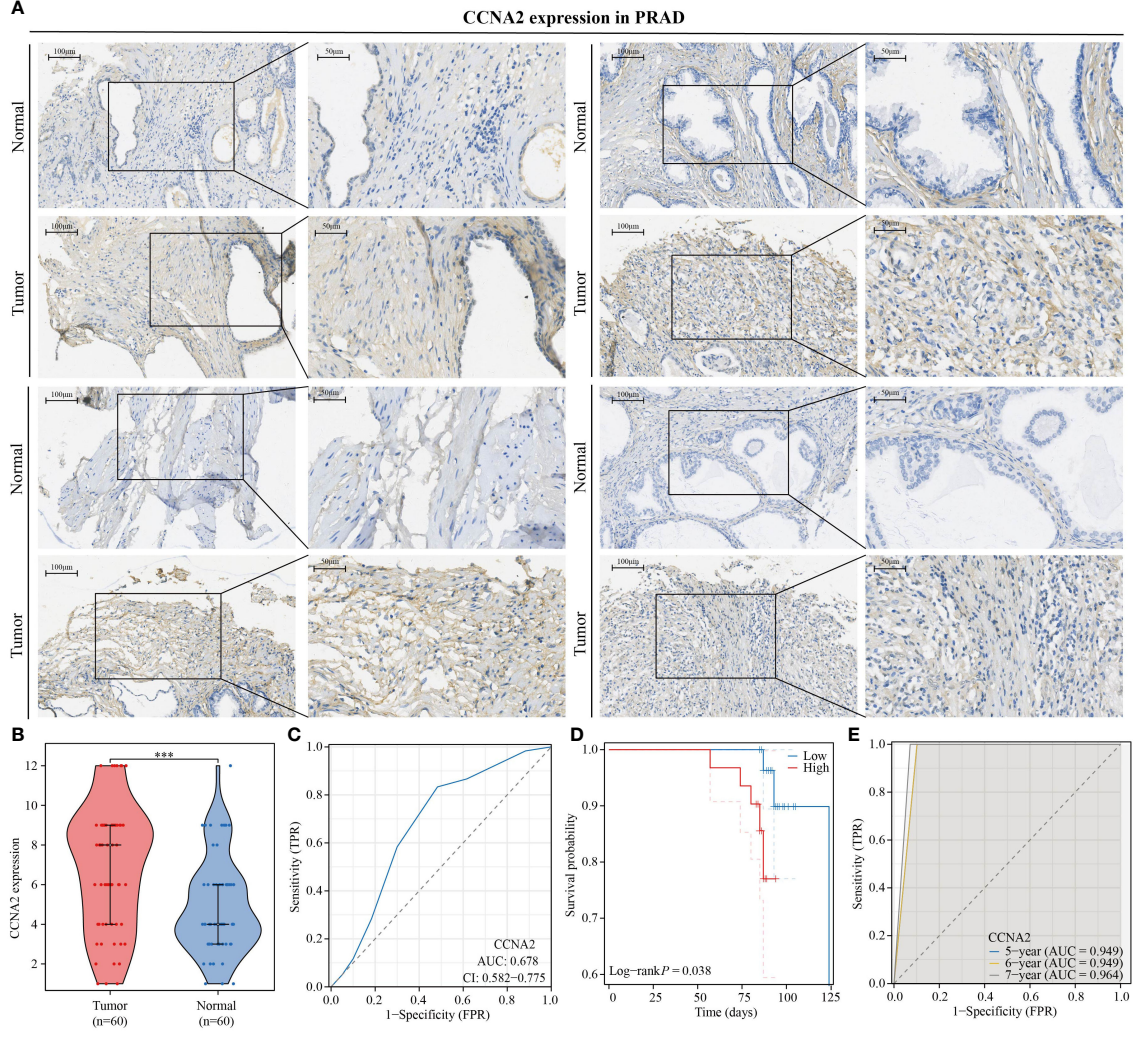
CCNA2 is highly expressed in PRAD and is associated with poor patient prognosis. **(A, B)** Differential expression of CCNA2 in PRAD. **(C)** Diagnostic predictive value of CCNA2 in PRAD. **(D)** KM curve of overall survival of CCNA2 in PRAD. **(E)** Prognostic predictive value of CCNA2 in PRAD.

In this study, we investigated the role of CCNA2 as a monocyte-related gene in PRAD. A total of 60 paired PRAD samples and corresponding paracancerous samples were collected for analysis of CCNA2 expression differences using immunohistochemical staining. Our results showed a significantly higher expression of CCNA2 in PRAD compared to normal tissues (**Figure 10A**). Violin plots were also utilized to visually represent the expression variances of CCNA2 in PRAD and normal tissues (**Figure 10B**). Furthermore, we assessed the diagnostic potential of CCNA2 for PRAD and found promising results, although further validation with larger sample sizes and clinical experiments is necessary (**Figure 10C**). Additionally, our analysis revealed a correlation between CCNA2 expression and the prognosis of PRAD patients, indicating a poorer prognosis for those with high CCNA2 expression levels (**Figure 10D**). Notably, CCNA2 showed strong predictive value for the prognosis of PRAD patients (**Figure 10E**). In conclusion, our experimental findings confirm the differential expression and prognostic implications of CCNA2 in PRAD.

## 4 Discussion

PRAD is a highly aggressive tumor with a poor prognosis, often being detected in advanced stages with metastasis (33). Biomarkers are essential in evaluating the therapeutic efficacy and prognosis of tumors and can be an essential component of precision medicine (34). Identifying PRAD and exploring new immune-related prognostic markers can significantly improve the efficacy of immunotherapy for individuals with this condition. The progression of tumors is closely connected to changes in the tumor microenvironment, where tumor cells impact their surroundings by releasing various chemokines and cytokines (35). Delving into the PRAD tumor microenvironment and discovering novel immune-related markers are essential for developing targeted therapeutic drugs and enhancing patient prognosis.

Research on monocytes in PRAD is increasing, with studies demonstrating their ability to stimulate PRAD cell invasion through pro-inflammatory cytokines (36). Circulating monocytes in metastatic PRAD patients have been found to secrete CHI3L1, promoting tumor growth (37). Our study revealed a correlation between higher levels of monocyte immune infiltration and poorer patient prognosis, aligning with previous findings on the carcinogenic role of monocytes. Through WGCNA analysis, we identified 14 monocyte-related genes. Among these genes, CCNA2, CDK1, CKS2, EZH2, HMGB3, KHDC4, KIF2C, PKMYT1, and PLK1 were found to be associated with various PRAD stages, Gleason scores, and PSA scores, further highlighting their significance in PRAD. Previous studies have also highlighted the importance of CCNA2 in PRAD using WGCNA analysis, but its prognostic value and correlation with monocytes have not been confirmed with clinical samples (38). CDK1 has been identified as a key player in promoting tumor metastasis in prostate cancer cells through its influence on the epithelial-mesenchymal transition process. This is achieved by regulating the phosphorylation of the ERK/GSK3β/SNAIL pathway (39). Additionally, CDK1 has been found to modulate the phosphorylation of the androgen receptor, with its inhibitors demonstrating the ability to enhance the effectiveness of enzalutamide in targeting prostate cancer cells (40, 41). Aberrant expression of CKS2 promotes prostate tumorigenesis by promoting proliferation and inhibiting programmed cell death (42). EZH2, HMGB3, KIF2C, PKMYT1 and PLK1 have also been confirmed to be related to PRAD progression (43–47). Subsequently, we developed a diagnostic model for monocyte-related genes using machine learning techniques. The Enet[alpha=0.4] method was the most recent approach employed to construct a diagnostic model for PRAD. Our analysis revealed that the diagnostic models built on the training set TCGA-PRAD and validation set GSE32571 demonstrated strong predictive value. However, in the validation set GSE62872, the AUC value was only 0.674, potentially influenced by the expression of TK1. Due to the imbalanced distribution of samples in the TCGA-PRAD data set, with only 10 patients deceased out of 498 samples, we opted to develop a prognostic model using the GSE16560 data set and validate it with the TCGA-PRAD data set. Our findings indicate that the prognostic model we created demonstrated robust predictive capabilities for the prognosis of PRAD patients, particularly at the 1-year and 7-year. GBM and RF algorithms were utilized to identify the genes most pertinent to PRAD prognosis within the monocyte-related genes. CCNA2 and ACSM3 were initially identified as the most relevant genes, but due to inconsistencies with prognostic correlation results in the TCGA-PRAD and GSE16560 datasets, ACSM3 was subsequently excluded. Ultimately, CCNA2 was identified as the gene most relevant to PRAD prognosis among the monocyte-related genes.

Single-cell genomics offers a novel approach to investigate the tumor immune microenvironment by conducting genomic analysis at the single-cell level. An increasing number of studies are utilizing this method to gain valuable insights (48–50). The correlation between CCNA2 and monocytes was examined using data from the TISCH2 websites. Our analysis showed a positive relationship between CCNA2 and monocyte infiltration levels. Additionally, CCNA2 was found to be positively associated with the expression of monocyte markers in PRAD. Through KEGG and GSEA analysis, we uncovered the significant role of CCNA2 in PRAD, potentially regulating cell senescence, apoptosis, and ferroptosis. Investigation into the correlation between CCNA2 and therapeutic drugs for PRAD revealed a strong binding affinity between CCNA2 and three specific drugs targeting PRAD. Moreover, CCNA2 exhibited strong binding capabilities with PD1 inhibitors, suggesting its potential as a drug targeting PRAD. However, our study is limited by a small sample size, which may have impacted our findings. It is essential to expand the sample size and validate these conclusions through further experimentation.

## 5 Conclusion

Our study highlighted the significant roles of monocyte-related genes in PRAD. Furthermore, we created and tested models utilizing different machine learning techniques to forecast the diagnosis and prognosis of PRAD patients. These results enhance our comprehension of monocyte infiltration patterns and underscore the significance of the monocyte-related gene CCNA2 as a valuable prognostic and diagnostic indicator for PRAD. These insights pave the way for personalized treatment strategies for patients with PRAD.

## Data availability statement

The raw data supporting the conclusions of this article will be made available by the authors, without undue reservation.

## Ethics statement

The studies involving humans were approved by Ethics Committee of Shanghai Outdo Biotech Company. The studies were conducted in accordance with the local legislation and institutional requirements. The participants provided their written informed consent to participate in this study. Written informed consent was obtained from the individual(s) for the publication of any potentially identifiable images or data included in this article.

## Author contributions

YW: Conceptualization, Data curation, Formal analysis, Validation, Writing – original draft. CL: Conceptualization, Data curation, Writing – original draft. JH: Data curation, Methodology, Writing – original draft. QZ: Software, Writing – original draft. YZ: Software, Writing – original draft. HS: Software, Writing – original draft. HZ: Validation, Writing – review & editing. BD: Funding acquisition, Writing – review & editing. MR: Funding acquisition, Project administration, Writing – review & editing.
